# Supplementary material for: CD4+ T cell-induced inflammatory cell death controls immune-evasive tumours
Source: Nature. 2023 Jun 14;618(7967):1033–40. doi: 10.1038/s41586-023-06199-x (PMC10307640; doi:10.1038/s41586-023-06199-x)
Supplement: Supplementary file 1 — This file contains Supplementary Fig. 1 and Table 1. Supplementary Fig. 1 Gating strategies used for flow cytometric analyses and cell sorting. Supplementary Table 1 sgRNA sequences used for CRISPR–Cas9-mediated cell engineering. [file 41586_2023_6199_MOESM1_ESM.pdf]

nature portfolio

https://doi.org/10.1038/s41586-023-06199-x

Supplementary information

CD4^{+} T cell-induced inflammatory cell death controls immune-evasive tumours

In the format provided by the authors and unedited

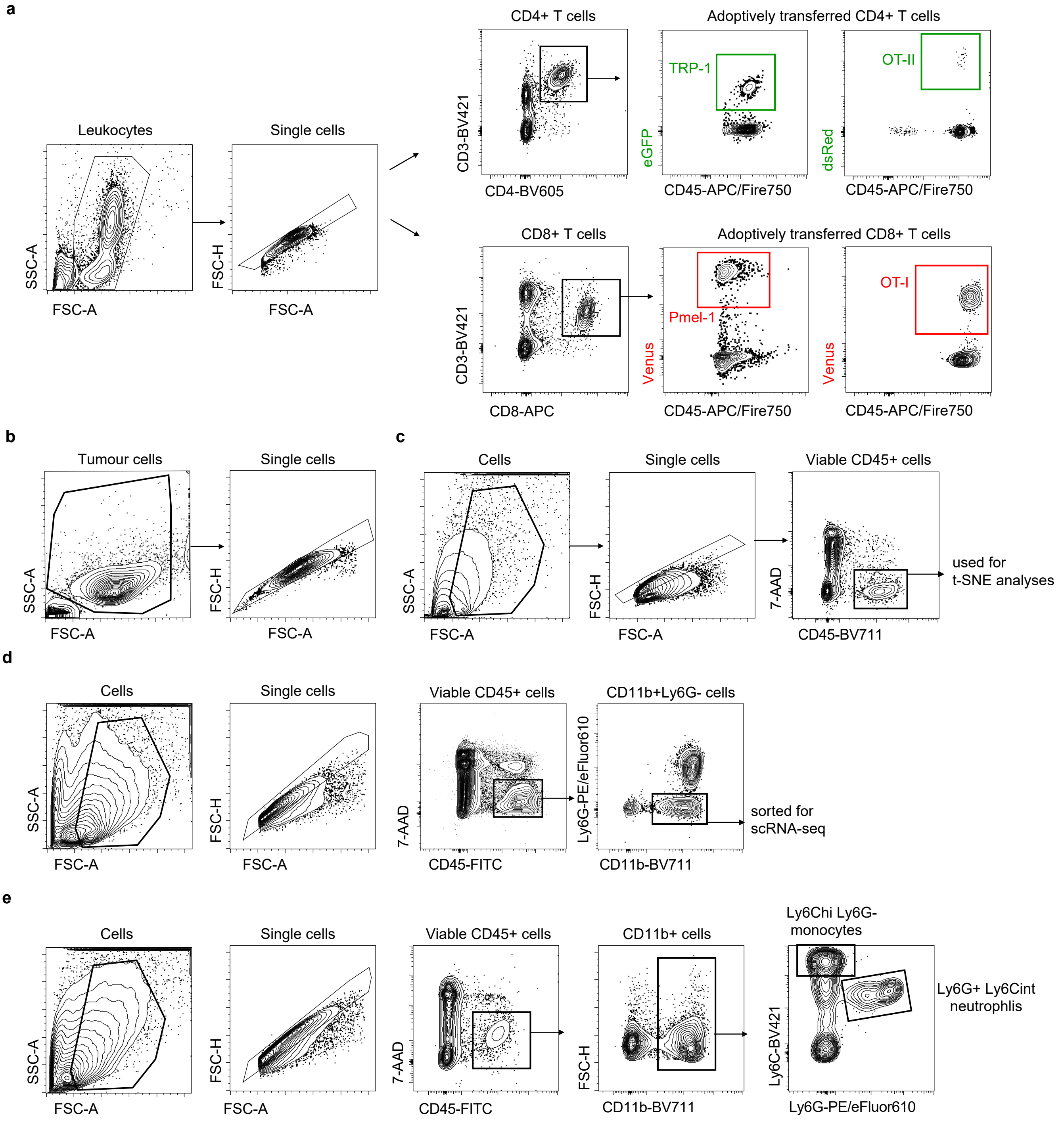

Supplementary Figure 1 │Gating strategies used for flow cytometric analyses and cell sorting.

a, Gating strategy to determine the percentage of adoptively transferred TRP-1, Pmel-1, OT-II or OT-I T cells out of all CD4+ or CD8+ T-cells, presented in Extended Data Fig. 2b and Extended Data Fig. 3g. **b,** Gating strategy to determine single tumour cells from in vitro cultures, used for further analysis on MHC-I and MHC-II expression, presented in Extended Data Fig. 2j and Extended data Fig. 3a, and cell death assays presented in Figure 4e,f and Extended Data Fig. 9g,h. **c,** Gating strategy to determine viable CD45+ immune cells used for subsequent t-SNE analyses, presented in Figure 1j,p; Figure 3f; Extended Data Fig. 2g,h, Extended Data Fig. 3d,e and Extended Data Fig. 7g,h. **d,** Gating strategy to sort viable CD45+CD11b+Ly6G- myeloid cells for single cell RNA-sequencing analyses presented in Figure 3a-e and Extended Data Fig. 7a-f. **e,** Gating strategy to determine the cells per mg tumour weight of Ly6ChiLy6G- monocytes and Ly6G+Ly6Cint neutrophils, presented in Extended Data Fig. 8b,g; Extended Data Fig. 9e, and the percentage of iNOS-expressing Ly6ChiLy6G- monocytes and Ly6G+Ly6Cint neutrophils, presented in Figure 3h,k and Extended Data Fig. 8b,g.

Supplementary Table 1: sgRNA sequences used for CRISPR/Cas9-mediated cell engineering

Guide sequence *Jak1* #1	TCCGTCTTGGGGTCCCGAAT
Guide sequence *Jak1* #2	CTCCGTCTTGGGGTCCCGAA
Guide sequence *Jak1* #3	GTTCTCGGCAATACGTTCAC
Guide sequence *Ciita* #1	GGGGGTCGGCATCACTGTTA
Guide sequence *Trp1* #1	GAGCCGCCATTATCCCCACGA
Guide sequence *Trp1* #2	GACTGTGGGACTTGCCGTCCT
Guide sequence *Trp1* #3	GAACTGTGGGACTTGCCGTCC
Guide sequence *Tyr* #1	GAATGCTGCCCACCATGGAT
