# Supplementary material for: CD4+ T cell-induced inflammatory cell death controls immune-evasive tumours
Source: Nature. 2023 Jun 14;618(7967):1033–40. doi: 10.1038/s41586-023-06199-x (PMC10307640; doi:10.1038/s41586-023-06199-x)
Supplement: Supplementary file 2 — Reporting Summary [file 41586_2023_6199_MOESM2_ESM.pdf]

nature portfolio

Corresponding author(s): Andreas J. Müller, Thomas Tüting

Last updated by author(s): Apr 26, 2023

Reporting Summary

Nature Portfolio wishes to improve the reproducibility of the work that we publish. This form provides structure for consistency and transparency in reporting. For further information on Nature Portfolio policies, see our Editorial Policies and the Editorial Policy Checklist.

Statistics

For all statistical analyses, confirm that the following items are present in the figure legend, table legend, main text, or Methods section.

n/a	Confirmed	
☐	☒	The exact sample size (*n*) for each experimental group/condition, given as a discrete number and unit of measurement
☐	☒	A statement on whether measurements were taken from distinct samples or whether the same sample was measured repeatedly
☐	☒	The statistical test(s) used AND whether they are one- or two-sided<br>*Only common tests should be described solely by name; describe more complex techniques in the Methods section.*
☐	☒	A description of all covariates tested
☐	☒	A description of any assumptions or corrections, such as tests of normality and adjustment for multiple comparisons
☐	☒	A full description of the statistical parameters including central tendency (e.g. means) or other basic estimates (e.g. regression coefficient) AND variation (e.g. standard deviation) or associated estimates of uncertainty (e.g. confidence intervals)
☐	☒	For null hypothesis testing, the test statistic (e.g. *F*, *t*, *r*) with confidence intervals, effect sizes, degrees of freedom and *P* value noted<br>*Give P values as exact values whenever suitable.*
☒	☐	For Bayesian analysis, information on the choice of priors and Markov chain Monte Carlo settings
☒	☐	For hierarchical and complex designs, identification of the appropriate level for tests and full reporting of outcomes
☒	☐	Estimates of effect sizes (e.g. Cohen's *d*, Pearson's *r*), indicating how they were calculated

Our web collection on statistics for biologists contains articles on many of the points above.

Software and code

Policy information about availability of computer code

Data collection	Attune NxT, Axio Imager.M2, LaVision TrimScope, Zeiss ZSM700 and Illumina Novaseq.
Data analysis	Graphpad Prism v8, Imaris v8.3 to 9.7, Flowjo v10.8.1, ImageJ 1.52i.<br><br>Python (version 3.9.9)<br>anndata (version 0.7.6)<br>anndata2ri (version 1.0.6)<br>attrs (version 21.2.0)<br>backcall (version 0.2.0)<br>certifi (version 2020.12.5)<br>cffi (version 1.14.5)<br>chardet (version 4.0.0)<br>CITE-seq-Count (version 1.4.4)<br>cmake (version 3.22.0)<br>cutadapt (version 3.5)<br>cycler (version 0.10.0)<br>Cython (version 0.29.24)<br>decorator (version 4.4.2)<br>get-version (version 2.2)<br>gprofiler-official (version 1.0.0)<br>h5py (version 3.2.1)<br>idna (version 2.10)<br>iniconfig (version 1.1.1)

ipykernel (version 5.5.4)
ipython (version 7.23.1)
ipython-genutils (version 0.2.0)
jedi (version 0.18.0)
Jinja2 (version 2.11.3)
joblib (version 1.0.1)
jupyter-client (version 6.1.12)
jupyter-core (version 4.7.1)
kiwisolver (version 1.3.1)
legacy-api-wrap (version 1.2)
llvmlite (version 0.36.0)
MarkupSafe (version 1.1.1)
matplotlib (version 3.4.2)
matplotlib-inline (version 0.1.2)
natsort (version 7.1.1)
networkx (version 2.5.1)
numba (version 0.53.1)
numexpr (version 2.7.3)
numpy (version 1.21.4)
openpyxl (version 3.0.9)
packaging (version 20.9)
pandas (version 1.2.4)
parso (version 0.8.2)
patsy (version 0.5.1)
pexpect (version 4.8.0)
pickleshare (version 0.7.5)
Pillow (version 8.2.0)
pluggy (version 0.13.1)
prompt-toolkit (version 3.0.18)
ptyprocess (version 0.7.0)
py (version 1.10.0)
pycparser (version 2.20)
Pygments (version 2.9.0)
pynndescent (version 0.5.2)
pyparsing (version 2.4.7)
python-dateutil (version 2.8.1)
pytz (version 2021.1)
pyxlsb (version 1.0.9)
pyzmq (version 22.0.3)
requests (version 2.25.1)
rpy2 (version 3.4.2)
scanpy (version 1.7.2)
scikit-learn (version 0.24.2)
scipy (version 1.6.3)
scvelo (version 0.2.4)
seaborn (version 0.11.1)
sinfo (version 0.3.4)
six (version 1.16.0)
statsmodels (version 0.12.2)
stdlib-list (version 0.8.0)
tables (version 3.6.1)
threadpoolctl (version 2.1.0)
toml (version 0.10.2)
tornado (version 6.1)
tqdm (version 4.60.0)
traitlets (version 5.0.5)
tzlocal (version 2.1)
umap-learn (version 0.5.1)
urllib3 (version 1.26.4)
wcwidth (version 0.2.5)
xlrd (version 1.2.0)
python-igraph (version 0.9.1)
leidenalg (version 0.8.4)
pytest (version 6.2.3)

R (version 4.0.4)
scran (version 1.18.7)
MAST (version 1.16.0)
SingleCellExperiment (version 1.12.0)
RcppAnnoy (version 0.0.16)
SummarizedExperiment (version 1.20.0)
Biobase (version 2.50.0)
GenomicRanges (version 1.42.0)
GenomeInfoDb (version 1.26.7)
IRanges (version 2.24.1)
S4Vectors (version 0.28.1)

BiocGenerics (version 0.36.1)
MatrixGenerics (version 1.2.1)
matrixStats (version 0.63.0)
gam (version 1.22)
foreach (version 1.5.1)
slingshot (version 1.8.0)
princurve (version 2.1.6)
glmnet (version 4.1-6)
Matrix (version 1.3-2)
RColorBrewer (version 1.1-3)
plyr (version 1.8.8)
ggplot2 (version 3.4.0)

For manuscripts utilizing custom algorithms or software that are central to the research but not yet described in published literature, software must be made available to editors and reviewers. We strongly encourage code deposition in a community repository (e.g. GitHub). See the Nature Portfolio guidelines for submitting code & software for further information.

Data

Policy information about availability of data

All manuscripts must include a data availability statement. This statement should provide the following information, where applicable:

- Accession codes, unique identifiers, or web links for publicly available datasets
- A description of any restrictions on data availability
- For clinical datasets or third party data, please ensure that the statement adheres to our policy

The raw sequencing mouse scRNA-seq data are available at the NCBI GEO under the accession GSE230427 without restrictions. The normalized and logarithmised count matrix used for the subsequent analyses is also available at the NCBI GEO under the accession GSE230427 without restrictions.
Human scRNAseq data used in this study are available at the European Genome-Phenome Archive (EGA) with the identifier EGAS00001006488, available for non-commercial research purposes upon reasonable request and subject to review of a project proposal that will be evaluated by the VIB-UZL Data Access Committee.

Field-specific reporting

Please select the one below that is the best fit for your research. If you are not sure, read the appropriate sections before making your selection.

☒ Life sciences ☐ Behavioural & social sciences ☐ Ecological, evolutionary & environmental sciences

For a reference copy of the document with all sections, see nature.com/documents/nr-reporting-summary-flat.pdf

Life sciences study design

All studies must disclose on these points even when the disclosure is negative.

Sample size	The determination of sample sizes for animal experiments was based on our experience with success of tumour engraftment and efficacy of therapeutic intervention in order to adhere to the 3R guidelines of the local Ethics Committee of the Office for Veterinary Affairs. Tumour treatment experiments involved 4-6 mice per group and were performed at least twice. Analyses of tumour immune cell infiltrates and intravital microscopy experiments involved a minimum of 3 mice per group. This yielded consistently reproducible and statistically significant results. Similarly, group sizes for in vitro experiments were determined based on prior knowledge of variation.
Data exclusions	No data was excluded from analysis.
Replication	Experiments were reliably reproduced and the number of experiments performed stated in methods and legends. Culminated and pooled data are shown where possible. Where representative data is shown, relevant experiments were repeated successfully at least twice with the exact number of repeats indicated in each case. Most experiments were repeated at least twice if not three or more times to verify that experimental findings were reproducible.
Randomization	For in vivo tumour treatment experiments, mice were randomized into different groups when the tumours reached between 3-5 mm in diameter.
Blinding	Blinding was not performed in this study. The experimental observations presented would be consistent irrespective of blinding and therefore blinding was not relevant in this study.

Reporting for specific materials, systems and methods

We require information from authors about some types of materials, experimental systems and methods used in many studies. Here, indicate whether each material, system or method listed is relevant to your study. If you are not sure if a list item applies to your research, read the appropriate section before selecting a response.

Materials & experimental systems

n/a	Involved in the study
☐	☒ Antibodies
☐	☒ Eukaryotic cell lines
☒	☐ Palaeontology and archaeology
☐	☒ Animals and other organisms
☐	☒ Human research participants
☒	☐ Clinical data
☒	☐ Dual use research of concern

Methods

n/a	Involved in the study
☒	☐ ChIP-seq
☐	☒ Flow cytometry
☒	☐ MRI-based neuroimaging

Antibodies

Antibodies used

MOUSE

Flow cytometry (Antibody, Supplier, Clone, Colour (Catalogue #) Dilution, Lot No.)

Anti-mouse CD45, Biolegend, 30-F11, APC Fire 750 (Cat #103154) 1:1600, B226658
Anti-mouse CD11c, Biolegend, N418, APC (Cat #117310) 1:200, B206713
Anti-mouse F4/80, Thermo Fisher, BM8, PE (Cat #12-4801-82) 1:300, 4299805
Anti-mouse CD11b, Biolegend, M1/70, BV711 (Cat #101242) 1:200, B379696
Anti-mouse Ly6C, Biolegend, HK1.4, PE-Cy7 (Cat #128018) 1:2000, B200606
Anti-mouse CD45R (B220), Biolegend, RA3-682, PE (Cat #103208) 1:1000, B224683
Anti-mouse CD3ε, Biolegend, 17A2, BV421 (Cat #100228) 1:500, B295089
Anti-mouse CD4, BD Biosciences, RM4-5, BV605 (Cat #563151) 1:500, 8039838
Anti-mouse NK1.1, Biolegend, PK136, APC (Cat #108710) 1:400, B191787
Anti-mouse CD45, BD Biosciences, 30-F11, FITC (Cat # 553079) 1:1000, 0030912
Anti-mouse F4/80, Biolegend, BM8, APC (Cat #123116) 1:200, B205476
Anti-mouse Ly6C, Biolegend, HK1.4, BV421 (Cat #128031) 1:800, B284703
Anti-mouse iNOS, Thermo Fisher, CXNFT, PE (Cat #12-5920-80) 1:300, 2283975
Anti-mouse I-A/I-E, Biolegend, M5/114.15.2, BV510 (Cat #107635) 1:800, B336985
Anti-mouse CD45, Biolegend, 30-F11, BV711 (Cat #103147) 1:200, B339309
Anti-mouse CD11c, Biolegend, N418, APC Fire 750 (Cat #117352) 1:100, B367888
Anti-mouse Siglec H, Biolegend, 551, FITC (Cat #129603) 1:400, B292161
Anti-mouse CD4, Thermofisher GK1.5, PE (Cat #12-0041-82) 1:1600, E01010-1635
Anti-mouse CD11b, Biolegend, M1/70, PE-Cy7 (Cat #101216) 1:2000, B203625
Anti-mouse Ly6G, BD Biosciences, 1A8, PE (Cat #551461) 1:800, 4246573
Anti-mouse CD3ε, Biolegend, 145-2C11, BV711 (Cat #100349) 1:100, B275433
Anti-mouse CD8α, Biolegend, 53-6.7, APC Fire 750 (Cat #100766) 1:1600, B247625
Anti-mouse H2-Kb, Biolegend, AF6-88.5, PE (Cat #116508) 1:500, B179854
Anti-mouse I-A/I-E, Biolegend, M5/114.15.2, APC (Cat #107614) 1:2000, B191785
Anti-mouse CD3ε, Biolegend, 145-2C11, FITC (Cat #100306) 1:100, B241616
Anti-mouse CD335 (NKp46), Biolegend, 29A1.4, APC (Cat #137608) 1:100, B375108
Anti-mouse CD8α, BD Biosciences, 53-6.7, PE (Cat #137608) 1:800, 5047674
Anti-mouse Vβ14 T cell receptor, BD Biosciences, 14-2, FITC (Cat # 553258) 1:2000, 6259505
Anti-mouse T-bet, Biolegend, 4B10, PeCy7, (Cat# 644824) 1:200, B214294
Anti-mouse Foxp3, Biolegend, MF-14, Alexa Fluor 647 (Cat# 126408) 1:100, B358685
Anti-mouse CD16/32, BioLegend, 93 (Cat # 101320) 1:300, B266362

Western blot (Antibody, Supplier, Clone (Catalogue #) Dilution, Lot No.)

Anti-mouse β-Actin (C4), Santa Cruz Biotechnology (Cat #sc-47778 HRP) 200 µg/ml, L3112
Anti-mouse TRP1 (M-19), Santa Cruz Biotechnology, Goat polyclonal (Cat #sc-10448) 1:1000, 0593-100808W5
Anti-goat HPR, Santa Cruz Biotechnology (Cat #sc-2354) 1:2000, A0319

In vivo depletion (Antibody, Supplier, Clone (Catalogue #) Lot No.)

Anti-mouse MHC-II, BioXCell, Y3P (Cat #BE0178), 796422M2
Anti-mouse NK1.1, BioXCell, PK136 (Cat #BE0036), 796521N1
Anti-mouse CD8, BioXCell, 2.43 (Cat #BE0061), 666418M1
Anti-mouse Ly6G, BioXCell, 1A8 (Cat #BE0075-1), 673218J1
Anti-mouse IFNg, BioXCell, XMG1.2 (Cat #BE0055), 791321M1
Anti-mouse CCR2, Matthias Mack, MC21

Single cell RNA sequencing hashtags (Antibody, Supplier, Clone (Catalogue #) Dilution, Lot No.)

TotalSeq™-B0301 anti-mouse Hashtag 1, Biolegend, M1/42; 30-F11, (Cat # 155831), 1:300, B324862
TotalSeq™-B0302 anti-mouse Hashtag 2, Biolegend, M1/42; 30-F11, (Cat # 155833), 1:300, B329819
TotalSeq™-B0303 anti-mouse Hashtag 3, Biolegend, M1/42; 30-F11, (Cat # 155835), 1:300, B324863
TotalSeq™-B0304 anti-mouse Hashtag 4, Biolegend, M1/42; 30-F11, (Cat # 155837), 1:300, B327527
TotalSeq™-B0305 anti-mouse Hashtag 5, Biolegend, M1/42; 30-F11, (Cat # 155839), 1:300, B318761

TotalSeq™-B0306 anti-mouse Hashtag 6, Biolegend, M1/42; 30-F11, (Cat # 155841), 1:300, B319551
TotalSeq™-B0307 anti-mouse Hashtag 7, Biolegend, M1/42; 30-F11, (Cat # 155843), 1:300, B326966
TotalSeq™-B0308 anti-mouse Hashtag 8, Biolegend, M1/42; 30-F11, (Cat # 155845), 1:300, B318319
TotalSeq™-B0309 anti-mouse Hashtag 9, Biolegend, M1/42; 30-F11, (Cat # 155847), 1:300, B326544
TotalSeq™-B03010 anti-mouse Hashtag 10, Biolegend, M1/42; 30-F11, (Cat # 155849), 1:300, B318317

Immunofluorescence (Antibody, Supplier, Colour, (Catalogue #) Dilution, Lot No.)

Rat anti-mouse I-A/I-E, BD Bioscience, M5/114.15.2, Purified (Cat #556999) 1:50, 6104526
Donkey anti-rat IgG (H+L), Jackson ImmunoResearch, Alexa Fluor 594 (Cat #712-585-150) 1:100, 126246

HUMAN

Immunohistochemistry (Antibody, Supplier, Clone (Catalogue #) Dilution, Lot No.)

Anti-human MHC-I (HLA-Class 1 ABC), Abcam, EMR8-5 (Cat #ab70328), 1:100, 20064861
Anti-human MHC-II (HLA-DP,DQ,DR), Abcam, CR3/43 (Cat #ab7856), 1:200, 12253498
Anti-human CD8, VENTANA, SP57 (Cat #05937248001), Undiluted, J16713
Anti-human MART-1 (MelanA), VENTANA, A103, (Cat #05278350001), Undiluted, J29957
Anti-human gp100, VENTANA, HMB45, (Cat #05479282001), Undiluted J27017
Anti-human S100, VENTANA, 4C4.9, (Cat #05278104001), Undiluted, J27878
Anti-human SOX10, Vitro Master Diagnostica, EP268 (Cat #MAD-000656QD-12), Undiluted, 06560046S

MILAN (Antibody, Supplier, Clone (Catalogue #) Dilution, Lot No.)

Anti-human CD3, Sigma Aldrich, polyclonal (Cat# C7930) 1 μg/mL, WB3189161
Anti-human panCK, Santa Cruz Biotechnology, LP5K (Cat# sc-53264) 1 μg/mL, 11246817
Anti-human CD4, Abcam, EPR6855, (Cat# ab133616) 1:200, GR3276764-17
Anti-human Foxp3, Abcam, 236A/E7, (Cat# ab20034) 1 μg/mL, GR3409148-10
Anti-human MHC-II, Novus Biologicals, SPM288 (Cat# NBP2-45312) 1 μg/mL, G0615
Anti-human CD68, Thermo Fischer Scientific, PGM1 (Cat# MA5-12407) 1:200, VB2949567
Anti-human Melan-A, Novus Biologicals, A19-P (Cat# NBP1-30151) 1:500, 41343161
Anti-human CD31, Santa Cruz Biotechnology, JC70 (Cat# sc-53411) 1 μg/mL, D1913
Anti-human CD11c, Santa Cruz, ITGAX (Cat# SC-46677), 1 μg/mL, H2416
Anti-human MITF, Dako, DS (Cat#M3621), 1 μg/mL, 10051273

Validation

All antibodies were obtained from commercial vendors and specificity was based on descriptions and information provided in corresponding data sheets provided by the manufacturers, and confirmed via in-house antibody titrations.

Validation statement for each antibody is provided on the manufacturer's website:

MOUSE

Flow cytometry

Anti-mouse CD45-APC Fire 750
https://www.biolegend.com/nl-be/products/apc-fire-750-anti-mouse-cd45-antibody-13049
Anti-mouse CD11c-APC
https://www.biolegend.com/de-at/products/apc-anti-mouse-cd11c-antibody-1813
Anti-mouse F4/80-PE
https://www.thermofisher.com/antibody/product/F4-80-Antibody-clone-BM8-Monoclonal/12-4801-82
Anti-mouse CD11b-BV711
https://www.biolegend.com/en-us/products/brilliant-violet-711-anti-mouse-human-cd11b-antibody-7927?GroupID=BLG10552
Anti-mouse Ly6C-PeCy7
https://www.biolegend.com/en-gb/products/pe-cyanine7-anti-mouse-ly-6c-antibody-6063
Anti-mouse CD45R, (B220) - PE
https://www.biolegend.com/de-de/products/pe-anti-mouse-human-cd45r-b220-antibody-447
Anti-mouse CD3ε -BV421
https://www.biolegend.com/de-de/products/brilliant-violet-421-anti-mouse-cd3-antibody-7326
Anti-mouse CD4-BV605
https://www.bdbiosciences.com/en-de/products/reagents/flow-cytometry-reagents/research-reagents/single-color-antibodies-ruo/bv605-rat-anti-mouse-cd4.563151
Anti-mouse NK1.1-APC
biolegend.com/en-us/products/apc-anti-mouse-nk-1-1-antibody-427?GroupID=GROUP20
Anti-mouse CD45-FITC
https://www.bdbiosciences.com/zh-cn/products/reagents/flow-cytometry-reagents/research-reagents/single-color-antibodies-ruo/fitc-rat-anti-mouse-cd45.553079
Anti-mouse F4/80-APC
https://www.biolegend.com/en-us/products/apc-anti-mouse-f4-80-antibody-4071?GroupID=BLG5319
Anti-mouse Ly6C-BV421
https://www.biolegend.com/de-de/products/brilliant-violet-421-anti-mouse-ly-6c-antibody-8586
Anti-mouse iNOS-PE
https://www.thermofisher.com/antibody/product/iNOS-Antibody-clone-CXNFT-Monoclonal/12-5920-82
Anti-mouse I-A/I-E-BV510
https://www.biolegend.com/en-us/products/brilliant-violet-510-anti-mouse-i-a-i-e-antibody-7997?GroupID=BLG11931

Anti-mouse CD45-BV711
https://www.biolegend.com/nl-nl/products/brilliant-violet-711-anti-mouse-cd45-antibody-10439
Anti-mouse CD11c-APC Fire 750
https://www.biolegend.com/en-us/products/apc-fire-750-anti-mouse-cd11c-antibody-13050?6664
Anti-mouse Siglec H- FITC
https://www.biolegend.com/nl-be/products/fitc-anti-mouse-siglec-h-antibody-5177
Anti-mouse CD4-PE
https://www.thermofisher.com/antibody/product/CD4-Antibody-clone-GK1-5-Monoclonal/12-0041-82
Anti-mouse CD11b-PE-Cy7
https://www.biolegend.com/en-us/products/pe-cyanine7-anti-mouse-human-cd11b-antibody-1921?GroupID=BLG10427
Anti-mouse Ly6G-PE
https://www.bdbiosciences.com/en-eu/products/reagents/flow-cytometry-reagents/research-reagents/single-color-antibodies-ruo/pe-rat-anti-mouse-ly-6g.551461
Anti-mouse CD3ε,-BV711
https://www.biolegend.com/fr-fr/products/brilliant-violet-711-anti-mouse-cd3epsilon-antibody-11975
Anti-mouse CD8α-APC Fire 750
https://www.biolegend.com/de-de/products/apc-fire-750-anti-mouse-cd8a-antibody-13048
Anti-mouse H2-Kb-PE
https://www.biolegend.com/en-us/products/pe-anti-mouse-h-2kb-antibody-1749?GroupID=BLG2539
Anti-mouse I-A/I-E-APC
https://www.biolegend.com/en-us/products/apc-anti-mouse-i-a-i-e-antibody-2488
Anti-mouse CD3ε-FITC
https://www.biolegend.com/en-us/products/fitc-anti-mouse-cd3epsilon-antibody-23
Anti-mouse CD335 (NKp46) - APC
https://www.biolegend.com/de-at/products/apc-anti-mouse-cd335-nkp46-antibody-6676?GroupID=BLG8849
Anti-mouse CD8α-PE
https://www.bdbiosciences.com/en-de/products/reagents/flow-cytometry-reagents/research-reagents/single-color-antibodies-ruo/pe-rat-anti-mouse-cd8a.553032
Anti-mouse Vβ14 T cell receptor-FITC
https://www.bdbiosciences.com/en-eu/products/reagents/flow-cytometry-reagents/research-reagents/single-color-antibodies-ruo/fitc-rat-anti-mouse-v-14-t-cell-receptor.553258
Anti-mouse T-bet-PeCy7
https://www.biolegend.com/en-us/products/pe-cyanine7-anti-t-bet-antibody-8328?GroupID=BLG6433
Anti-mouse Foxp3-Alexa Fluor 647
https://www.biolegend.com/en-us/products/alexa-fluor-647-anti-mouse-foxp3-antibody-4662
Anti-mouse CD16/32
https://www.biolegend.com/nl-be/products/trustain-fcx-anti-mouse-cd16-32-antibody-5683

Western blot

Anti-mouse TRP1
https://datasheets.scbt.com/sc-10448.pdf
Anti-mouse β-Actin
https://datasheets.scbt.com/sc-47778.pdf
Anti-goat HPR
https://www.scbt.com/p/mouse-anti-goat-igg-hrp

In vivo depletion

Anti-mouse MHC-II
https://bioxcell.com/invivomab-anti-mouse-mhc-class-ii-i-a-be0178
Anti-mouse NK1.1
https://bioxcell.com/invivomab-anti-mouse-nk1-1-be0036
Anti-mouse CD8
https://bioxcell.com/invivomab-anti-mouse-cd8a-be0061
Anti-mouse Ly6G
https://bioxcell.com/invivomab-anti-mouse-ly6g
Anti-mouse IFNg
https://bioxcell.com/invivomab-anti-mouse-ifng-be0055
Anti-mouse CCR2
Mack et al., 2001 Journal of Immunology

Single cell RNA sequencing hashtags
TTotalSeq™-B0301 anti-mouse Hashtag 1
https://www.biolegend.com/en-us/products/totalseq-b0301-anti-mouse-hashtag-1-antibody-17771
TotalSeq™-B0302 anti-mouse Hashtag 2
https://www.biolegend.com/en-us/products/totalseq-b0302-anti-mouse-hashtag-2-antibody-17772
TotalSeq™-B0303 anti-mouse Hashtag 3
https://www.biolegend.com/en-us/products/totalseq-b0303-anti-mouse-hashtag-3-antibody-17773
TotalSeq™-B0304 anti-mouse Hashtag 4
https://www.biolegend.com/en-us/products/totalseq-b0304-anti-mouse-hashtag-4-antibody-17774
TotalSeq™-B0305 anti-mouse Hashtag 5
https://www.biolegend.com/en-us/products/totalseq-b0305-anti-mouse-hashtag-5-antibody-17775
TotalSeq™-B0306 anti-mouse Hashtag 6
https://www.biolegend.com/en-us/products/totalseq-b0306-anti-mouse-hashtag-6-antibody-17776
TotalSeq™-B0307 anti-mouse Hashtag 7

https://www.biolegend.com/en-us/products/totalseq-b0307-anti-mouse-hashtag-7-antibody-17777
TotalSeq™-B0308 anti-mouse Hashtag 8
https://www.biolegend.com/en-us/products/totalseq-b0308-anti-mouse-hashtag-8-antibody-17778
TotalSeq™-B0309 anti-mouse Hashtag 9
https://www.biolegend.com/en-us/products/totalseq-b0309-anti-mouse-hashtag-9-antibody-17779
TotalSeq™-B03010 anti-mouse Hashtag 10
https://www.biolegend.com/en-us/products/totalseq-b0310-anti-mouse-hashtag-10-antibody-18225

Immunofluorescence

Rat anti-mouse I-A/I-E-Purified
https://www.bdbiosciences.com/en-us/products/reagents/flow-cytometry-reagents/research-reagents/single-color-antibodies-ruo/purified-rat-anti-mouse-i-a-i-e.556999
Donkey anti-rat IgG (H+L)
https://www.jacksonimmuno.com/catalog/products/712-585-150

HUMAN

Immunohistochemistry

Anti-MHC-I (HLA-Class 1 ABC)
https://www.abcam.com/products/primary-antibodies/hla-class-1-abc-antibody-emr8-5-ab70328.html
Anti-MHC-II (HLA-DP,DQ,DR)
https://www.abcam.com/products/primary-antibodies/hla-dr--dp--dq-antibody-cr343-ab7856.html
Anti-human CD8
https://shop.roche-diagnostics.ch/labor/05937248001
Anti-human MART-1 (MelanA)
https://shop.roche-diagnostics.ch/labor/05278350001
Anti-human gp100
https://shop.roche-diagnostics.ch/labor/05479282001
Anti-human S100
https://shop.roche-diagnostics.ch/labor/05278104001
Anti-human SOX10
https://www.medac-diagnostika.de/index.php?controller=product&id_product=10566

MILAN

Anti-human CD3
https://www.sigmaaldrich.com/DE/en/product/sigma/c7930
Anti-human panCK
https://www.scbt.com/p/cytokeratin-7-antibody-lp5k
Anti-human CD8
https://www.thermofisher.com/antibody/product/CD8-Antibody-clone-SP16-Monoclonal/MA5-16345
Anti-human CD4
https://www.abcam.com/products/primary-antibodies/cd4-antibody-epr6855-ab133616.html
Anti-human Foxp3
https://www.abcam.com/products/primary-antibodies/foxp3-antibody-236ae7-ab20034.html
Anti-human MHC-II
https://www.novusbio.com/products/hla-drb1-antibody-spm288_nbp2-45312
Anti-human CD68
https://www.thermofisher.com/antibody/product/CD68-Antibody-clone-PG-M1-Monoclonal/MA5-12407
Anti-human MLANA
https://www.novusbio.com/products/melan-a-mart-1-antibody-a19-p_nbp1-30151
Anti-human CD31
https://www.scbt.com/p/pecam-1-antibody-jc70
Anti-human CD11c
https://www.scbt.com/p/integrin-alphax-antibody-b-6
Anti-human MITF
https://www.agilent.com/en/product/immunohistochemistry/antibodies-controls/primary-antibodies/mitf-(concentrate)-76592#productdetails

Eukaryotic cell lines

Policy information about cell lines

Cell line source(s)

The mouse HCmel12 cell line and all variants were generated in the Tüting Laboratory. The mouse B16 melanoma cell line was purchased from ATCC.

The human melanoma cell lines MaMel04 and MaMel102 were kindly provided by Dirk Schadendorf. The human melanoma cell lines, Skmel28 and A375, and HEK293T cells were purchased from ATCC. The 911 human embryonic retinoblast cell line was obtained from Crucell.

Authentication

B16, Skmel28, A375 and HEK293T cells were originally obtained from ATCC respectively and were therefore authenticated by

Authentication	the manufacturer. Furthermore, all cell lines were subjected to STR fingerprinting analysis. Successful gene knock-out for the CRISPR-variants of HCmel12 were all confirmed on a genomic (next-generation sequencing), on a transcriptomic (q-PCR)/proteomic (western blot) and functional level. Fluorescence was always confirmed by flow cytometry.
Mycoplasma contamination	Cell lines regularly tested negative for mycoplasma contamination.
Commonly misidentified lines (See ICLAC register)	No commonly misidentified cell lines were used in this study.

Animals and other organisms

Policy information about studies involving animals; ARRIVE guidelines recommended for reporting animal research

Laboratory animals	Mice were housed in an ambient temperature- and humidity-controlled environment on a 12-hour light/dark cycle to mimic natural conditions. Laboratory mouse (Mus musculus) strains C57BL/6J mice were purchased from Janvier or Charles River. Pmel-1, TRP1, OT-I and OT-II mice were purchased from Jackson Laboratories and bred in Central Animal Laboratory, House 65, University Hospital Magdeburg. Pmel-1-Venus mice were generated by crossing CAG-Venus mice with pmel-1 mice. TRP-1-eGFP mice were generated by crossing B6-eGFP mice into the TRP-1-deficient Rag1-KO background of TRP-1 mice. OT-I-Venus mice were generated by crossing CAG-Venus mice with OT-I mice. OT-II-dsRed were generated by crossing OT-II mice with hCD2-dsRed mice (kindly provided by Cornelia Harlin). Pmel-Venus, TRP1-GFP, OT-I-Venus, OT-II-dsRed and CD11c-Venus mice were bred in Central Animal Laboratory, House 65, University Hospital Magdeburg. All transgenic strains were maintained on a C57BL/6 background. All mice were aged between 8 and 12 weeks of age at the time experiments commenced. All animal experiments were conducted with male mice on the C57BL/6 background under specific pathogen-free conditions in individually ventilated cages according to the institutional and national guidelines for the care and use of laboratory animals.
Wild animals	No wild animals were used in the study.
Field-collected samples	No field collected samples were included in the study.
Ethics oversight	Approval by the Ethics Committee of the Office for Veterinary Affairs of the State of Saxony-Anhalt, Germany (permit license numbers 42502-2-1393 Uni MD, 42502-2-1586 Uni MD, 42502-2-1615 Uni MD and 42502-2-1672 Uni MD) in accordance with legislation of both the European Union (Council Directive 499 2010/63/EU) and the Federal Republic of Germany (according to § 8, Section 1 TierSchG, and TierSchVersV).

Note that full information on the approval of the study protocol must also be provided in the manuscript.

Human research participants

Policy information about studies involving human research participants

Population characteristics	Melanoma metastases of 12 male and 8 female patients with a median age of 76 years (range 35-88 years) were biopsied in the Department of Dermatology at the Univeristy Hospital Magdeburg. Melanoma metastases of 9 male and 11 female patients with a median age of 66 years (range 34-82 years) were biopsied the Department of Oncology at the UZ Leuven.
Recruitment	From the University Hospital Magdeburg, samples from melanoma metastases were collected as part of a non-interventional single-centre study investigating the dynamics of the inflammatory immune cell composition.<br><br>From UZ Leuven, biopsies from melanoma metastases were collected as part of a non-interventional single-center prospective study investigating transcriptomic changes upon immune checkpoint inhibition (Prospective Serial biopsy collection before and during immune-checkpoint inhibitor therapy in patients with malignant melanoma (SPECIAL). Biopsies were taken from easily accessible sites (skin, subcutis, lymph node).
Ethics oversight	Participants from the University Hospital Magdeburg: Ethical approval for the observational study under the title "Dynamics of inflammatory responses during the initiation and progression of skin cancer"(Study No. 162/20).<br><br>Participants from UZ Leuven: Ethical approval from the UZ Leuven Medical Ethical Committee.

Note that full information on the approval of the study protocol must also be provided in the manuscript.

Flow Cytometry

Plots

Confirm that:

☒ The axis labels state the marker and fluorochrome used (e.g. CD4-FITC).

☒ The axis scales are clearly visible. Include numbers along axes only for bottom left plot of group (a 'group' is an analysis of identical markers).

☒ All plots are contour plots with outliers or pseudocolor plots.

☒ A numerical value for number of cells or percentage (with statistics) is provided.

Methodology

Sample preparation	Blood samples were resuspended in red cell lysis buffer and incubated for 15 minutes at room temperature. The samples were then centrifuged at 350g for 5 minutes and the supernatant was discarded. This process was repeated. FC-Blocking was performed by incubation of the samples with anti-CD16/32 (1:300) for 10 minutes at 4°C. After washing the samples, the cells were then stained with antibodies for 15 minutes at 4°C. The samples were subsequently washed and resuspended in FACS buffer prior to analysis.<br><br>Tumours, spleens and lymph nodes were homogenised through a 70 µm strainer to generate single cell suspensions. The samples were then centrifuged at 350g for 5 minutes and the supernatant was discarded. The samples were then resuspended in red cell lysis buffer and incubated for 5 minutes at room temperature. The samples were then centrifuged again and the supernatant was discarded prior to FC-Blocking of the samples with anti-CD16/32 (1:300) for 10 minutes at 4° C. After washing the samples, the cells were then stained with antibodies for 15 minutes at 4°C. The samples were subsequently washed and resuspended in FACS buffer prior to analysis.
Instrument	Attune NxT flow cytometer.
Software	Attune NxT for collection and Flowjo v10.8.1 (Treestar) for analysis.
Cell population abundance	To quantify the abundance of immune cell subpopulations in tumour tissues, 2000 cells of interest per biological sample were concatenated to a single FCS file. t-SNE plots were generated in FlowJo using the opt-SNE learning configuration (https://www.nature.com/articles/s41467-019-13055-y). The vantage-point tree KNN algorithm and the Barnes-Hut gradient algorithm set to 1000 iterations, 30 perplexity and 840 learning rate.
Gating strategy	Please refer to Supplementary Figure 1.<br>For blood, Pmel cells were identified using the following gating strategy: FSCH lo/SSCH intermediate (lymphocytes) --> FSCA/FSCWlo (singlet gate) --> +/- CD45+ (lymphocytes) --> CD8+ Venus (transgenic Pmel).<br>For blood, Trp1 cells were identified using the following gating strategy: FSCH lo/SSCH intermediate (lymphocytes) --> FSCA/FSCWlo (singlet gate) --> +/- CD45+ (lymphocytes) --> CD8+ eGFP+(transgenic Trp1).<br>For blood, OT.I cells were identified using the following gating strategy: FSCH lo/SSCH intermediate (lymphocytes) --> FSCA/FSCWlo (singlet gate) --> +/- CD45+ (lymphocytes) --> CD8+ Venus+ (transgenic OT.I).<br>For blood, OT.II cells were identified using the following gating strategy: FSCH lo/SSCH intermediate (lymphocytes) --> FSCA/FSCWlo (singlet gate) --> +/- CD45+ (lymphocytes) --> CD4+ dsRED+ (transgenic OT.II).<br>To assess cell death, melanoma cells were identified using the following gating strategy: FSCA/SCCA --> FSCA/FSCWlo (singlet gate) --> PI+ Annexin+ (dead cells) .<br>To quantitate MHC expression, melanoma cells were identified using the following gating strategy: FSCA/SCCA --> FSCA/FSCW lo (singlet gate) --> MHC-I hi or MHC-II hi (histogram gate).<br>To phenotype intratumoural CD4+ T cells in Figure 1: FSCH lo/SSCH intermediate (lymphocytes) --> FSCA/FSCW lo (singlet gate) --> +/- CD45+ (lymphocytes) --> CD3+ CD4+ --> eGFP+ (transferred) --> T-bet+ (Th1), Foxp3+ (Treg).<br>To quantitate immune subsets in Figure 1 and Extended Data Figure 3, leukocytes were identified using the following gating strategy: FSCA/SSCA --> FSCA/FSCWlo (singlet gate) --> CD45+ 7AAD- (live leukocytes) --> Immature monocytes (CD11b+ Ly6C hi), mature macrophages, (CD11b+ F4/80+), mature monocytes (CD11b+ Ly6C lo), TRP1 CD4 (GFP+), dendritic cells (CD11b+ MHC-II+ CD11c+), endogenous lymphocytes (CD11b- CD11c-), neutrophils (CD11b+ Ly6G+).<br>To quantitate immune subsets in Figure 1 and Extended Data Figure 5, leukocytes were identified using the following gating strategy: FSCA/SSCA --> FSCA/FSCWlo (singlet gate) --> CD45+ 7AAD- (live leukocytes) --> Immature monocytes (CD11b+ Ly6C hi), mature macrophages (CD11b+ F4/80+), mature monocytes (CD11b+ Ly6c lo), TRP1 CD4 (GFP+), Pmel CD8 (Venus+), dendritic cells (CD11b+ MHC-II+ CD11c+ F4/80-), endogenous lymphocytes (CD11b- CD11c-), neutrophils (CD11b+ Ly6G+).<br>To quantitate immune subsets in Figure 3 and Extended Data Figure 9, leukocytes were identified using the following gating strategy: FSCA/SSCA --> FSCA/FSCW lo (singlet gate) --> CD45+ 7AAD- (live leukocytes) --> Immature monocytes (CD11b+ Ly6C hi), mature macrophages (CD11b+ F4/80+ iNOS-), iNOS+ mono/macs (CD11b+ Ly6C hi iNOS+) mature monocytes (CD11b+ Ly6c lo), dendritic cells (CD11b+ MHC-II+ CD11c+ F4/80-), endogenous lymphocytes (CD11b- CD11c-), neutrophils (CD11b+ Ly6G+).<br>To quantitate immune subsets in Extended Data Figure 10, leukocytes were identified using the following gating strategy: FSCA/SSCA --> FSCA/FSCWlo (singlet gate) --> CD45+ 7AAD- (live leukocytes) --> Monocytes (CD11b+ Ly6C hi) and neutrophils (CD11b+ Ly6G+).

☒ Tick this box to confirm that a figure exemplifying the gating strategy is provided in the Supplementary Information.
